# Prevalence of classical swine fever in Karnataka, India

**DOI:** 10.14202/vetworld.2015.541-544

**Published:** 2015-04-29

**Authors:** Prakash Choori, S. S. Patil, D. Rathnamma, R. Sharada, B. M. Chandranaik, S. Isloor, G. B. Manjunath Reddy, S. Geetha, H. Rahman

**Affiliations:** 1 Department of Veterinary Microbiology, Veterinary College, KVAFSU, Hebbal, Bengaluru, Karnataka, India; 2 Department of Virology, National Institute of Veterinary Epidemiology and Disease Informatics (Formerly PD_ADMAS), Hebbal, Bengaluru, Karnataka, India; 3 Department of Microbiology, Institute of Animal Health and Veterinary Biologicals, Hebbal, Bengaluru, Karnataka, India

**Keywords:** antigen, antibody, classical swine fever, ELISA and prevalence

## Abstract

**Aim:**

The present study was conducted to know the current scenario of classical swine fever (CSF) in Bengaluru Urban, Bengaluru Rural, Chikkaballapur, Madikeri, Mandya, Bagalkot, Gadag, Yadgir, Koppal, and Bidar districts of Karnataka with the using of both antigen and antibody ELISA.

**Materials and Methods:**

We collected 218 sera and 121 blood samples from pigs from 10 different districts of Karnataka. Screening of sera for CSF IgG antibody and whole blood for CSF virus antigen were carried out using the CSF virus (CSFV) antibody and antigen ELISA kits, respectively.

**Results:**

The mean seroprevalence was 41% (89/218) and prevalence of CSFV antigen in blood samples was 32% (39/121) for the 10 districts of Karnataka. Seroprevalence of 61%, 29%, 20%, and 21%; and antigen prevalence of 40%, 50%, 13%, and 12% were recorded for Bangalore, Mysore, Belgaum, and Gulbarga divisions of Karnataka, respectively.

**Conclusions:**

The study revealed an alarmingly high prevalence of CSF, both for the antigen (32%) and antibody (41%) in Karnataka. Southern Karnataka has the highest seroprevalence (61% in Bangalore and 29% in Mysore divisions), which confirms the endemicity of the disease in that region. This could be attributed to the intensive pig farming practices in the region as compared to Northern Karnataka (Seroprevalence of 20% in Belgaum and 21% in Gulbarga divisions), where the commercial pig farming is still in infantile stages.

## Introduction

Classical swine fever (CSF) is a highly contagious disease of swine that has caused major economic losses in industrialized pig producing countries around the world [1]. CSF virus (CSFV), the causative agent of CSF, is a member of the genus *Pestivirus*, which belongs to the family Flaviviridae. CSF is a World Organization for Animal Health (OIE) listed disease and outbreaks are reportable, with resultant trade sanctions against the affected countries [2]. CSF is one of the top five viral diseases of livestock in India (foot and mouth disease, bluetongue, peste des petits ruminants, sheep and goat pox, CSF) and is a major constraint to the development of pig farming in the country [3]. The disease has been successfully eradicated from Canada (1963) and the United States of America (1976), and has been under effective control within European Union in recent years [4]. However, the situation of recurrent CSF epidemics in Asia, Latin America, East Europe, and former USSR area is still serious. In Asia, especially, the reported cases in 2004 increased by nearly 20% when compared to 2003. Recently, outbreaks re-emerged in South Africa, which had been free of CSF since 1918 [5]. CSF is highly endemic in adjoining countries like China [6], and there are recent reports of the disease from Nepal [7] and Bhutan [8]. As with other OIE listed diseases, many countries have insufficient resources to undertake adequate surveillance. This, along with political and economic pressures which tend to shift focus from disease surveillance, and the masking effect of vaccination, are likely to result in an under-reporting of the true extent of the disease worldwide [2].

The first documented report of CSF in India dates back to 1962, where an outbreak in a piggery unit in Morol, a suburb of Mumbai (formerly Bombay), and later in other parts of the city were described [9]. There are also reports of outbreaks of the disease by Krishnamurthy and Adlakha [10], Damodaran *et al*. [11] and Saini *et al*. [12] from the states of Uttar Pradesh, Tamil Nadu, and Punjab, respectively. A compilation of data from OIE website indicates that there were 1308 outbreaks of CSF in India during 1996-2008 [13]. The disease is also most frequently reported from Karnataka due to considerable density of pigs in the state [14].

Because of the sporadic nature of the disease and the lower preference for the pig farming (barring North-Eastern states) in India, CSF has not been studied systematically and therefore epidemiology of the disease is largely not fully understood [15]. Occurrence of CSFV genotype 1.1 and more recently dominance of genotype 2.2 were documented in Karnataka [13,16] and there are reports that the currently dominating phylogenetic Group 2 has been replaced the historical groups (1.1, 1.2 and 1.3) [17].

However, there are not much data available on the seroprevalence of CSFV infection in Karnataka. The present study was undertaken to obtain the baseline epidemiological information on the prevalence of CSF by analyzing both antigen and antibody in whole blood and serum, respectively.

## Materials and Methods

### Ethical approval

Institutional Animal Ethics Committee of Veterinary College, KVAFSU, Hebbal, Bangalore has accorded permission for the collection of blood from pigs for the study.

### Sampling and test procedure

**Figure-1:**
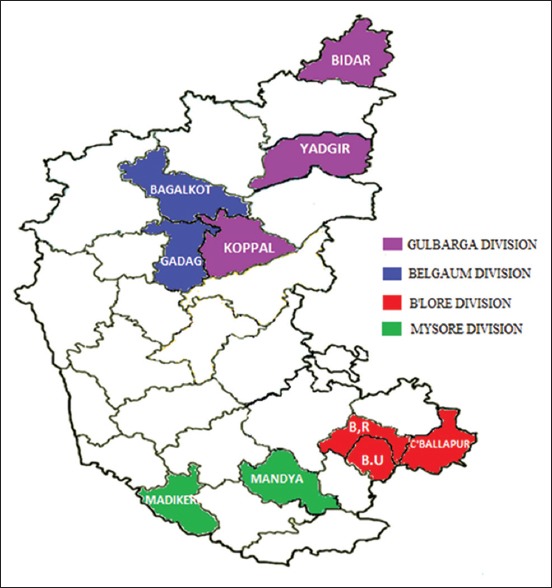
Map showing the various divisions and districts of Karnataka (shaded in different colors) from where samples were collected.

Blood (n=121) and serum samples (n=218) were collected from pigs inappropriate vacutainer tubes from 10 different districts of Karnataka during September, 2013 to July, 2014 (Figure-1). Clotted blood samples were spun at 1100-1300 rpm for 15 min, sera separated and stored at 20°C until tested. Screening of whole blood for CSF antigen and sera for CSF antibody was carried out using the CSFV antigen and antibody ELISA kits (IDEXX Laboratories, Netherlands), respectively. For antigen ELISA, samples were considered positive when the corrected absorbance values were ≥0.30, negative when <0.20, and doubtful between 0.20 and 0.30, requiring re-test ing. In antibody ELISA testing, samples were considered negative when the blocking percentage was ≤30% and positive when ≥40%. The samples were considered suspicious if the values were between 30% and 40%, and were retested at later date.

### Statistical analysis

Univariate descriptive statistical analysis was used to calculate the percent positivity of antigen prevalence, seroprevalence and also to draw the significant inference.

## Results and Discussion

The study was conducted to determine the prevalence of CSF in Karnataka. The mean prevalence of CSFV antigen in blood samples was 32% (39/121) and seroprevalence was 41% (89/218) for the 10 districts of Karnataka. Prevalence by antigen ELISA was shown to be 40%, 50%, 13%, and 12% and seroprevalence was 61%, 29%, 20%, and 21% for Bangalore, Mysore, Belgaum, and Gulbarga divisions of Karnataka, respectively (Tables-1 and 2).

**Table-1:** Seroprevalence of classical swine fever in different regions of Karnataka.

Division	Districts	Location	Number of serum samples collected	Number of samples positive by antibody ELISA	Percent positivity
Bangalore	Bangalore Urban	Aagara	08	02	25.00
		Kuduregere	17	12	70.58
		Somashettyhalli	19	10	52.63
	Bangalore Rural	Thathanur	07	07	100
		Hosallipalya	17	17	100
		Navaratna Agrahara	23	11	47.82
	Chikkaballapur	Bagepalli	12	04	33.33
	Total		103	63	61.16*
Mysore	Madikeri	Kadagadalu	13	05	38.46
	Mandya	Doddarasinakere	11	02	18.18
	Total		24	07	29.16*
Belgaum	Bagalkot	Bevoor	24	04	16.66
	Gadag	Mundaragi	20	05	25.00
	Total		44	09	20.45
Gulbarga	Yadgir	Kodekal	22	07	31.81
	Koppal	Hanamasagar	21	00	00
	Bidar	Chintaki	04	03	75.00
	Total		47	10	21.27
Total			218	89	40.82

* Statistically significant (p<0.01)

**Table-2:** Prevalence of classical swine fever by antigen ELISA in different regions of Karnataka.

Division	Districts	Location	Number of Blood samples collected	Number of samples positive by Antigen ELISA	Percent positivity
Bangalore	Bangalore Urban	Aagara	08	05	62.50
		Kuduregere	14	04	28.57
		Somashettyhalli	10	01	10.00
	Bangalore Rural	Thathanur	06	03	50.00
		Hosallipalya	11	05	45.45
		Navaratna Agrahara	08	05	62.50
	Chikkaballapur	Bagepalli	-	-	-
	Total		57	23	40.35*
Mysore	Madikeri	Kadagadalu,	13	07	53.84
	Mandya	Doddarasinakere	09	04	44.44
	Total		22	11	50.00*
Belgaum	Bagalkot	Bevoor	16	02	12.50
	Gadag	Mundaragi	-	-	-
	Total		16	02	12.50
Gulbarga	Yadgir	Kodekal	13	02	15.38
	Koppal	Hanamasagar	13	01	07.69
	Bidar	Chintaki	-	-	-
	Total		26	03	11.53
Total			121	39	32.23

* Statistically significant (p<0.01)

The results of samples from Bangalore and Mysore divisions were found to be statistically significant (p<0.01) when compared to samples from Belgaum and Gulbarga divisions. The incumbent changes were seen in samples from Bangalore and Mysore divisions both for antigen and seroprevalence.

During 2014, a CSF study conducted in Karnataka showed an overall 33% (173/517) and 43%, 33%, 12%, and 6% of seroprevalences recorded for Bangalore, Mysore, Gulbarga, and Belgaum divisions, respectively [18].

An alarmingly high prevalence of CSF antigen (32%) and antibodies (41%) in Karnataka, and notable prevalence of disease in the northern region where the piggery is still in primitive stages, was revealed. The regional distribution of antigen and seroprevalence reveals that Southern Karnataka (40% and 61% in Bangalore respectively, and 50% and 29% in Mysore divisions accordingly) has the highest CSF prevalence, confirming the endemicity of the disease in that region. The Northern part of Karnataka showed a lower antigen and seroprevalence when compared to the South (13% and 20% in Belgaum, respectively, and 12% and 21% in Gulbarga divisions accordingly).

The high prevalence of CSF in the southern region was attributed to a large number of pig farms when compared to the North, and regular procurement of piglets from pig breeding farms located in the adjoining border areas of Tamil Nadu and Kerala, where there were no CSF vaccination campaigns. The low prevalence of the disease in North Karnataka is probably because of the presence of less organized pig farms in the region, due to non-consumption of pork by the residents for social and cultural reasons.

The study conducted at National Institute of Veterinary Epidemiology and Disease Informatics, Bangalore during 2012-2013 showed the seroprevalence of CSF in Kerala and Tamil Nadu to be approximately 77% (31/40) and 100% (10/10), respectively [14]. This was probably due to the high prevalence of CSF in these states, which spread to neighboring South Karnataka due to procurement of piglets, resulting in the observed high antigen and seroprevalences. Nandi *et al*. [19] reported CSF seroprevalence of 63% from 12 different states of India during 2004-2010 and 53% prevalence in Southern India alone.

Sero-surveillance, especially using techniques such as ELISA, is highly useful in disease eradication programs as it helps for mass screening and is effective in early diagnosis in nearby regions of an outbreak and becomes a good tool for surveillance of negative herds in countries where monitoring of the disease is being practiced. Prevalence based on the presence of viral antigen in the sample is generally used to estimate the incidence of the disease and when a non-vaccinated herd is positive for CSF-specific antibodies, it is can be considered as an indicative of a CSF disease outbreak. The present study confirms the prevalence of the disease in various regions of Karnataka as it allowed screening of samples representing all the areas of the state.

## Conclusions

The study revealed an alarmingly high prevalence of CSF, both for the antigen (32%) and antibody (41%) in Karnataka. Southern Karnataka has the highest seroprevalence (61% in Bangalore and 29% in Mysore divisions), which confirms the endemicity of the disease in that region. This could be attributed to the intensive pig farming practices in the region as compared to Northern Karnataka (Seroprevalence of 20% in Belgaum and 21% in Gulbarga divisions), where the commercial pig farming is still in infantile stages.

## Authors’ Contributions

SSP and DR designed the experiment. PC carried out the study along with BMC and SG. RS and GBMR analyzed the data and prepared the manuscript. SI and HR reviewed the manuscript. All authors participated in scientific discussion. All authors read and approved the final manuscript.

## References

[1] Moennig V, Floegel-Niesmann G, Greiser-Wilke I (2003). Clinical signs and epidemiology of classical swine fever: A review of new knowledge. Vet. J.

[2] Paton D.J, Greiser-Wilke I (2003). Classical swine fever – An update. Res. Vet. Sci.

[3] Patil S.S, Hedadri D, Veeresh H, Sreekala K, Gajendragad M.R, Prabhudas K (2012). Phylogenetic analysis of NS5B gene of classical swine fever virus isolates indicated plausible Chinese origin of Indian subgroup 2.2 viruses. Virus Genes.

[4] Dong X.N, Chen Y.H (2007). Marker vaccine strategies and candidate CSFV marker vaccines. Vaccine.

[5] OIE Disease Information dated 15 July (2005). http://web.oie.int/eng/info/hebdo/AIS_60.HTM.

[6] Luo Y, Li S, Sun Y, Qiu HJ (2014). Classical swine fever in China: A minireview. Vet. Microbiol.

[7] Postel A, Jha V.C, Schmeiser S, Becher P (2013). First molecular identification and characterization of classical swine fever virus isolates from Nepal. Arch. Virol.

[8] Monger V.R, Stegeman J.A, Koop G, Dukpa K, Tenzin T, Loeffen W.L.A (2014). Seroprevalence and associated risk factors of important pigviral diseases in Bhutan. Prev. Vet. Med.

[9] Sapre S.N, Moghe R.G, Bhagwat S.V, Chaudhry P.G, Purohit B.L (1962). A note on observations and investigations into an outbreak of swine fever in Bombay (Maharashtra). Indian Vet. J.

[10] Krishnamurthy D, Adlakha S.C (1962). A preliminary report on the swine fever epidemic in Uttar Pradesh. Indian Vet. J.

[11] Damodaran S, Ramakrishnan R, Rahmathulakhan S (1971). Swine fever in Madras. Indian Vet. J.

[12] Saini S.S, Dhand N.K, Sharma D.R, Sood S.K (2000). An outbreak of swine fever in Punjab. Indian J. Vet. Pathol.

[13] Patil S.S, Hedadri D, Shankar B.P, Raghavendra A.G, Veeresh H, Sindhoora B, Chandan S, Sreekala K, Gajendragad M.R, Prabhudas K (2010). Genetic typing of recent classical swine fever isolates from India. Vet. Micobiol.

[14] Patil S. S, Hemadri D, PD_ADMAS (2013). Annual Report 2012-13. Epidemiology of Classical Swine Fever in India.

[15] Sarma D.K, Bostami B (2008). Isolation and growth characteristics of classical swine fever in PK-15 cell line. J. Appl. Biosci. Biotechnol.

[16] Shivaraj D.B (2014). Molecular epidemiology and diagnosis of classical swine fever by reverse transcriptase polymerase chain reaction.

[17] Lin T, Li X, Yao H, Wei Z, Liu R, Deng Y, Zhai S, Li W, Sun L, Long J, Zhang H, Lu J, Yuan S (2013). Phylogenetic diversity of classical swine fever virus (CSFV) field isolates from outbreaks in China between 2008 and 2011. Asian J. Anim. Vet. Adv.

[18] Shivaraj D.B, Patil S.S, Rathnamma D, Veeregowda B.M, Hemadri D, Geetha S, Reddy G.B.M, Sharada R, Shesharao P, Rahman S (2013). Seroepidemiology of classical swine fever in Karnataka. Indian J. Field Vet.

[19] Nandi S, Muthuchelvan D, Ahuja A, Bisht S, Chander V, Pandey A.B, Singh R.K (2011). Prevalence of classical swine fever virus in India: A 6-Year study (2004-2010). Transbound. Emerg. Dis.

